# Current Status of a Model System: The Gene *Gp-9* and Its Association with Social Organization in Fire Ants

**DOI:** 10.1371/journal.pone.0007713

**Published:** 2009-11-06

**Authors:** Dietrich Gotzek, Kenneth G. Ross

**Affiliations:** 1 Department of Ecology and Evolution, University of Lausanne, Lausanne, Switzerland; 2 Department of Entomology, University of Georgia, Athens, Georgia, United States of America; University of Alabama, United States of America

## Abstract

The *Gp-9* gene in fire ants represents an important model system for studying the evolution of social organization in insects as well as a rich source of information relevant to other major evolutionary topics. An important feature of this system is that polymorphism in social organization is completely associated with allelic variation at *Gp-9*, such that single-queen colonies (monogyne form) include only inhabitants bearing *B*-like alleles while multiple-queen colonies (polygyne form) additionally include inhabitants bearing *b*-like alleles. A recent study of this system by Leal and Ishida (2008) made two major claims, the validity and significance of which we examine here. After reviewing existing literature, analyzing the methods and results of Leal and Ishida (2008), and generating new data from one of their study sites, we conclude that their claim that polygyny can occur in *Solenopsis invicta* in the U.S.A. in the absence of expression of the *b*-like allele *Gp-9^b^* is unfounded. Moreover, we argue that available information on insect OBPs (the family of proteins to which GP-9 belongs), on the evolutionary/population genetics of *Gp-9*, and on pheromonal/behavioral control of fire ant colony queen number fails to support their view that GP-9 plays no role in the chemosensory-mediated communication that underpins regulation of social organization. Our analyses lead us to conclude that there are no new reasons to question the existing consensus view of the *Gp-9* system outlined in Gotzek and Ross (2007).

## Introduction

The genetic and physiological foundations of insect social behavior increasingly are the subjects of study and, as a result, are becoming understood in ever greater detail [1]. One element of social behavior of intense interest in this regard is the number of reproductive queens in a colony, a basic component of colony social organization that often is linked to a host of other important reproductive and life-history traits in ants [2]–[4]. Regulation of colony queen number in fire ants (genus *Solenopsis*) has been the focus of considerable study over the past several decades. An important feature that has emerged from this work is that polymorphism in colony social organization in the red imported fire ant, *S. invicta*, is associated with variation at a single gene, *General protein-9* (*Gp-9*) [5], [6]. Specifically, colonies with a single mated reproductive queen (monogyne form) include only inhabitants bearing a class of *Gp-9* alleles referred to as *B*-like alleles; in contrast, colonies containing multiple mated reproductive queens (polygyne form) always additionally include inhabitants bearing an alternate class of alleles, designated *b*-like alleles (see Table 1) [5], [7]–[9]. Similar patterns of an apparently invariant association between colony social organization and *Gp-9* genotypic composition have been found in several close relatives of *S. invicta* [9], [10]. This consistent pattern has led to the conclusion that the presence in a fire ant colony of a minimal frequency of workers with *b*-like alleles (10–15%) is both necessary and sufficient to elicit polygyne social behavior [11], [12], suggesting that a profound distinction in colony social organization is under the control of a single Mendelian factor of large effect.

**Table 1:** Relevant terminology for *Gp-9* and colony social organization in fire ants.

**Alate queen:** winged, virgin adult ant queen that is not reproductively active.
***B*** **-like alleles:** paraphyletic assemblage of fire ant *Gp-9* alleles characterized by coding for three diagnostic amino acid residues (Ser^42^, Met^95^, Val^139^); in the socially polymorphic species, colonies of the monogyne social form possess only *B*-like alleles; three *B* allele haplotypes (*B1*, *B2*, *B3*) that differ only in their non-coding sequences have been reported previously from *S. invicta* in the U.S.A. [8].
***b*** **-like alleles:** monophyletic group of fire ant *Gp-9* alleles characterized by coding for three diagnostic, apomorphic amino acid residues (Gly^42^, Ile^95^, Ile^139^); in the socially polymorphic species, colonies of the polygyne social form possess *b*-like alleles along with *B*-like alleles.
***b*** ** alleles:** monophyletic subset of *b*-like alleles of *Gp-9* characterized by the apomorphic charge-changing Glu151Lys replacement; the only *b*-like allele previously reported from *S. invicta* in the U.S.A. (*b1*) is of this type [8] and is characterized also by encoding a diagnostic Ala^136^ residue.
***b′*** ** alleles:** paraphyletic subset of *b*-like alleles of *Gp-9* that lack the charge-changing Glu151Lys replacement.
**Dealate queen:** wingless adult ant queen generally assumed to be reproductively active; reproductive dealate queens in polygyne nests of *S. invicta* may or may not be mated [49].
***Gp-9*** **:** gene encoding GP-9 protein in fire ants and other *Solenopsis* species; *Gp-9* contains five exons and four introns and, in the true fire ants, is 1700 bp in length [8], [10].
**GP-9:** protein encoded by *Gp-9* composed of 153 amino acids in the native form (134 amino acids in the mature form; 14.7 kDa estimated molecular mass); GP-9 is a member of the insect OBP family [6].
**Insect odorant binding proteins (OBPs):** diverse family of extracellular carrier proteins generally characterized by their small size (∼15 kDa), presence of a signal sequence, and six cysteine residues arranged in a characteristic pattern; some members (the **“gold standard” OBPs**) have been implicated as component molecular transducers of chemical to neuronal signals—these proteins, shown to be confined to chemosensillar lymph and to bind relevant ligands [21], [55], are thought to function in insect chemosensilla by passively transporting exogenous, hydrophobic chemostimulants through the lymph to receptors on sensory neuron dendrites, by stimulating sensory neuron activity, by controlling extracellular chemostimulant concentration, and/or by sequestering chemostimulants after signal transduction [21], [22], [60]–[62], [65], [66].
**Monogyne social form:** form of *S. invicta* and other fire ant species in which colony social organization features a single mated reproductive queen.
**Polygyne social form:** form of *S. invicta* and other fire ant species in which colony social organization features multiple (two to many hundred) mated reproductive queens.

Given the unique aspects of this system, including the apparently simple genetic basis for a complex, emergent social phenotype, *Gp-9* in fire ants has become an important model for study of the genetic regulation and evolution of social organization [6], [13]–[15]. Additionally, results from this system have played into key debates on several other major topics of evolutionary significance, including the existence of selfish “green beard” genes [16], the evolutionary balance between selection and gene flow [5], [17], the operation of selection at different hierarchical levels [6], [18], and the importance of indirect genetic effects in social evolution [19], [20].

The product of *Gp-9* is a member of the insect odorant binding protein (OBP) family, a large and diverse family including some members that have demonstrated roles as transducers of chemical to neuronal stimuli within peripheral chemosensilla [21], [22] (see Table 1). This fact, combined with data from *S. invicta* showing that regulation of colony queen number involves discrimination among queens by workers based on specific chemical signals emanating from queens [6], [16], led to an early hypothesis of the functional role played by GP-9 in mediating social organization. This hypothesis can be summarized as follows: GP-9 functions in a manner similar to the “gold standard” OBPs (Table 1) implicated as molecular chemoreception transducers; queen-produced pheromones comprise ligands of GP-9; the B-like and b-like protein variants differ in their binding properties with respect to these ligands; workers of different *Gp-9* genotypes exhibit different queen recognition capabilities; and the different worker *Gp-9* genotype compositions in each form generate different colony-level phenotypes of collective worker tolerance toward queens [8], [23]. Several other scenarios involving specific biochemical and physiological role(s) of GP-9 in relation to regulation of social organization subsequently have been proposed [6], [24], [25], and the possibilities that *Gp-9* plays only an indirect, complementary, or effectively no role in mediating social organization also have been considered [6], [8], [26].

Given the extent and significance of the body of work on *Gp-9*, challenges to general conclusions that have emerged must be viewed seriously. Recently, Leal and Ishida [27; henceforth LI08] studied the expression of *Gp-9* in several colonies of *S. invicta* sampled in its introduced range in the U.S.A. Their experiments led them to two major findings, the first of which directly contradicts earlier conclusions and the second of which was anticipated in earlier work. First, LI08 failed to confirm expression of the *b*-like allele *Gp-9^b^* (the only *b*-like allele known from *S. invicta* in the U.S.A.; Table 1) in several putative polygyne colonies. Second, they confirmed that GP-9 protein is present in adult hemolymph and thus distributed throughout the body. From these findings, LI08 concluded that “…it is highly unlikely that GP-9s are involved in olfactory mediation of social organization of the red imported fire ant.” In this paper, we assess the methods and results of LI08, review important published data overlooked by these authors, and present new data relevant to their claims and conclusions. We find that their failure to detect GP-9^b^ protein in putative polygyne colonies is likely an artifact of flawed sampling design and experimental procedures. We further find that a failure of LI08 to properly evaluate and integrate previous relevant research not only led to their misrepresentation of the current state of knowledge but undermines their central conclusion regarding the role of *Gp-9* in controlling social organization in fire ants. Our analysis leads us to conclude that there are no new reasons to question the general consensus view of the *Gp-9* system as it stands [reviewed in 6], while prompting us to re-emphasize the need to pursue particular avenues of research designed to fill important gaps in our knowledge of this system.

## Analysis

### Horizontal Starch Gel Electrophoresis as a Tool for Studying GP-9

Most of the early research on *Gp-9* was conducted using horizontal starch gel electrophoresis (HSGE) coupled with non-specific amido black staining as a method for surveying variation in the protein product of the gene, and the method continues to serve as a workhorse for efficiently conducting assays for a variety of uses in GP-9 research. In reference to this work, LI08 state that “… [GP-9] gel profiles were not documented in the literature” and “…native polyacrylamide gels (PAGE) … provides more consistent and reproducible profiles…”. Because much of what appears in the remainder of the current paper is based on results from HSGE, we offer the following comments with respect to the quality, reproducibility, and utility of the technique.

**Figure 1:**
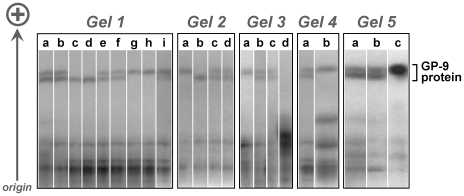
Separation and visualization of GP-9 protein in *S. invicta* using HSGE coupled with amido black staining. The GP-9 band of higher mobility is encoded by the *B* allele, whereas the band of lower mobility is encoded by the *b* allele. Material from which GP-9 was extracted and electrophoresed is as follows. *Gel 1*: lanes a–b, individual polygyne reproductive queen thoraces (both genotype *Bb*); lanes c–d, individual polygyne alate queen thoraces (both genotype *bb*); lanes e–f, individual polygyne alate queen thoraces (both genotype *Bb*); lanes g–h, individual polygyne alate queen thoraces (both genotype *BB*); lane i, individual triploid polygyne alate queen thorax (genotype *BBb*). *Gel 2*: lane a, individual polygyne alate queen head (genotype *BB*); lane b, individual polygyne alate queen head (genotype *bb*) ; lanes c–d, individual polygyne alate queen heads (both genotype *Bb*). *Gel 3*: lane a, individual adult worker head+thorax (genotype *BB*); lane b, individual adult worker head+thorax (genotype *Bb*); lane c, eggs (several hundred pooled) from polygyne nest (both B and b allelic proteins present); lane d, larvae (several hundred pooled 2nd instar) from polygyne nest (GP-9 not apparent). *Gel 4*: lane a, individual polygyne alate queen thorax (genotype *Bb*); lane b, monogyne adult male thoraces (50 pooled, only *B* allelic protein apparent). *Gel 5*: lane a, individual polygyne reproductive queen thorax (genotype *Bb*); lane b, individual polygyne reproductive queen hemolymph (genotype *Bb*); lane c, monogyne adult worker hemolymph (pooled from ten individuals, only *B* allelic protein apparent).

Details of gel and buffer compositions, running conditions, staining procedures, and absolute and relative protein migration distances for GP-9 using HSGE were provided a decade ago by DeHeer et al. [28]. Because HSGE separates proteins almost exclusively on the basis of net charge [29], [30], this technique is expected to distinguish only two classes of GP-9 proteins, those encoded by alleles of the *b* clade, which feature a charge-changing Glu151Lys replacement (Table 1), and those encoded by the remaining *Gp-9* alleles, which lack this replacement [8]–[10]. This expectation has been confirmed using a large number of parallel protein/DNA assays in several fire ant species, with the relative HSGE mobilities of the protein products of *Gp-9^b^* and the other alleles always as predicted from the inferred amino acid sequences [8], [10], [31] (e.g., Fig. 1). The fact that the derived, charge-variant *Gp-9^b^* alleles are the only *b*-like alleles known to occur in the U.S.A. [8] (see also below) has led to the use of HSGE as a reliable, high-throughput tool for detecting polygyny in large sample sets from these invasive populations [e.g.], [32,33].

We note that HSGE/GP-9 assays have been employed in several other contexts that, in aggregate, validate assay sensitivity and reproducibility. These assays were used: i) to predict the occurrence of *b*-like *Gp-9* alleles that lack the charge-changing amino acid replacement characteristic of *b* alleles (the former class designated as *b′* alleles; Table 1) in both *S. invicta* [26] and the related fire ant *S. richteri* [31], ii) to determine the ontogeny of expression of GP-9 in brood and adult females [5], as later confirmed using specific mRNA and polyclonal antibody assays [34, D. Gotzek et al. unpubl. data], iii) to confirm the presence of GP-9 at low levels in adult males [34] and in eggs [D. Gotzek et al. unpubl. data], as initially deduced using specific mRNA and antibody assays (see Fig. 1), and iv) to validate triploidy of *S. invicta* females, as confirmed in conjunction with microsatellite genotyping [35] (Fig. 1). Moreover, the equivalence of HSGE and SDS-PAGE coupled with amido black staining for separating and purifying GP-9 protein was demonstrated by Krieger and Ross [8; their Note 8 and Supplementary Online Material]. Although HSGE is conducted on pooled material (multiple individuals from single colonies) for some applications, the method is sufficiently sensitive to be used on individual adult queen heads and all but the smallest adult minor worker thoraces+heads [12], [36] (Fig. 1). Importantly, where we have judged HSGE to be inadequate to address particular questions, alternative molecular methods for assaying variation at the *Gp-9* gene and its product have been employed; these include allele-specific PCR [9], [12], [37], [38], restriction fragment length polymorphism (RFLP) analysis [8], [31], direct sequencing of cDNA and genomic DNA [8]–[10], [38], microarray expression assays [20], and specific mRNA and polyclonal antibody assays [D. Gotzek et al. unpubl. data].

### Expression of the *Gp-9^b^* Allele in Polygyne *S. invicta* Colonies

A central claim of LI08 is that there exist “…two types of polygyne colonies, one that does not express GP-9^b^ (monogyne-like) and the other expressing both proteins, GP-9^B^ and GP-9^b^.” This claim was based on the inability of the authors to detect the b allelic protein among workers (and apparently queens as well, see their Fig. 10) in three of the four ostensibly polygyne *S. invicta* colonies they studied. Such a finding hypothetically could be attributable either to a lack of workers in a polygyne colony bearing the *b* allele or to the inability of workers bearing the allele to express it (these possibilities were not distinguished nor discussed by LI08). To give a sense of the extraordinary nature of the claim that three-quarters of their polygyne study colonies lacked workers expressing GP-9^b^, we summarize below the published evidence supporting an invariant link between the expression of polygyne social organization and both the presence and expression of *b*-like *Gp-9* alleles in a fire ant colony's workers (and queens). We note that for *S. invicta* in the U.S.A., the association may be more narrowly defined as an invariant link between allele *Gp-9^b^* (or its product) and polygyny, assuming that this is the only *b*-like allele that occurs in this newly colonized part of the range [8] (see also below).

Fifteen studies have documented the presence of *b*-like alleles in all polygyne colonies examined (Table 2); these studies surveyed a total of 1259 polygyne colonies of five different fire ant species (mostly *S. invicta*) sampled from three continents. Only eight of these studies explicitly tested for the presence of this class of *Gp-9* alleles in the worker caste, but the remainder tested reproductive queens. All such queens possessed at least one *b*-like allele copy so, barring some unreported form of non-Mendelian inheritance of the gene [cf. 5], some proportion of workers in these study colonies can also be inferred to have possessed such alleles.

**Table 2:** Studies documenting invariant presence of *b*-like *Gp-9* alleles in inhabitants of polygyne fire ant colonies.

Study	Species	Geographic source of study colonies	*n*, *N* a	Comments
Ross (1997) [5]	*Solenopsis invicta*	Georgia and Texas, U.S.A.	1986, **245**	reproductive queens as source material, genotypes determined by HSGE
Ross and Keller (1998) [7]	*S. invicta*	Georgia, U.S.A.	124, **53**	reproductive queens as source material, laboratory units headed individually by 37 of these queens behaved like polygyne colonies, genotypes determined by HSGE
Goodisman et al. (1999) [36]	*S. invicta*	Georgia, U.S.A.	2226, **114**	adult workers as source material, sampled from 13 colonies maintained in the laboratory and from 101 field colonies, genotypes determined by HSGE
Krieger and Ross (2002) [8]	*S. invicta*	California, Georgia, Florida, and Texas, U.S.A.	13, **13**	reproductive queens as source material, HSGE genotyping confirmed by DNA sequencing
	*S. macdonaghi*	Argentina	1, **1**	reproductive queen as source material, genotype determined by DNA sequencing
	*S. quinquecuspis*	Argentina	1, **1**	reproductive queen as source material, genotype determined by DNA sequencing
	*S. richteri*	Argentina	1, **1**	reproductive queen as source material, genotype determined by DNA sequencing
Ross and Keller (2002) [11]	*S. invicta*	Georgia, U.S.A.	403, **11**	adult workers as source material, genotypes determined by HSGE
Valles and Porter (2003) [76]	*S. invicta*	Florida, U.S.A.	?, **20**	adult workers (pooled) and/or reproductive queens as source material, genotypes determined by allele-specific multiplex PCR
Krieger and Ross (2005) [10]	*S. megergates*	Brazil	1, **1**	reproductive queen as source material, genotype determined by DNA sequencing
Fritz et al. (2006) [77]	*S. invicta*	Florida, U.S.A.	516, **117**	reproductive queens as source material, genotypes determined by PCR/RFLP assay
Shoemaker et al. (2006) [32]	*S. invicta*	Georgia, Florida, eastern Louisiana, western Louisiana, Mississippi, and Texas, U.S.A.	4344, **543** b	adult workers and/or queens as source material, genotypes determined by HSGE
Goodisman et al. (2007) [78]	*S. invicta*	Georgia, U.S.A.	1139, **5**	adult workers as source material, genotypes determined by allele-specific multiplex PCR
Gotzek et al. (2007) [9]	*S. invicta*	Argentina, Brazil	95, **30**	adult workers and/or queens as source material, genotypes determined by DNA sequencing
Hallar et al. (2007) [31]	*S. richteri*	Argentina	79, **27**	reproductive queens as source material, genotypes determined by PCR/RFLP assay
Gotzek and Ross (2008) [12]	*S. invicta*	Georgia, U.S.A.	656, **15**	reproductive queens as source material, genotypes determined by HSGE
Wang et al. (2008) [20]	*S. invicta*	Georgia and Louisiana, U.S.A.	400, **20**	adult workers and/or reproductive queens as source material, HSGE genotyping confirmed by PCR/RFLP assay
Yang et al. (2008) [51]	*S. invicta*	Taiwan	>420^c^, **42**	adult workers as source material, genotypes determined by allele-specific multiplex PCR

Several of these studies [e.g.], [5], [9], [12], [20], [51,76] also showed that monogyne colonies lack *b*-like alleles. HSGE, horizontal starch gel electrophoresis; PCR/RFLP, polymerase chain reaction/restriction fragment length polymorphism.

a *n*, number of individuals sampled; ***N***, number of polygyne colonies from which these individuals were sampled.

b Four additional study colonies were classified as monogyne based on worker size and brood composition, absence of multiple reproductive queens, and absence of the *Gp-9^b^* allele, but evidence from allozyme genotype distributions suggested that each comprised multiple families; the discrepancy was explained as resulting from recent queen turnover in these monogyne colonies.

c 10–15 workers per nest were pooled and their DNA extracted in bulk.

Five studies have documented that the *b* allele not only is present but invariably is expressed in workers from polygyne *S. invicta* colonies in the introduced U.S.A. range (Table 3). This conclusion was based on the presence of the b allelic protein as assessed by HSGE in variable numbers of individual workers from all 225 colonies studied. Moreover, five studies have employed HSGE to show that, among thousands of reproductive queens sampled from many hundred polygyne colonies located across the U.S.A. range, each queen expressed the b allelic protein (Table 3).

**Table 3:** Studies documenting expression of *b* allele of *Gp-9* in adult female inhabitants of polygyne *S. invicta* colonies using HSGE.

Study	Material	Geographic source of study colonies	*n*, *N* a	Percentage of polygyne study colonies with individuals expressing *b* allele	Comments
Ross (1997) [5]	workers	Georgia, U.S.A.	1758, **60**	100	field colonies as sources
	reproductive queens	Georgia and Texas, U.S.A.	1986, **245**	100	field colonies as sources
Ross and Keller (1998) [7]	workers	Georgia, U.S.A.	400, **20**	100	workers sampled from laboratory units that were headed individually by polygyne reproductive queens and that behaved like polygyne colonies
	reproductive queens	Georgia, U.S.A.	87, **23**	100	laboratory colonies as sources
Goodisman et al. (1999) [36]	workers	Georgia, U.S.A.	2226, **114**	100	workers sampled from 13 laboratory colonies and 101 field colonies; proportions of workers expressing allele *Gp-9* b in each colony not reported in [36] but determined from original data
Goodisman et al. (2000b) [79]	reproductive queens	Georgia, U.S.A.	1183, **5** b	100	field colonies as sources
Ross and Keller (2002) [11]	workers	Georgia, U.S.A.	403, **11**	100	laboratory colonies as sources
Shoemaker et al. (2006) [32]	reproductive queens	Georgia, Florida, eastern Louisiana, western Louisiana, Mississippi, and Texas, U.S.A.	543, **543**	100	field colonies as sources
Gotzek and Ross (2008) [12]	reproductive queens	Georgia, U.S.A.	656, **15**	100	laboratory colonies as sources
Wang et al. (2008) [20]	workers	Georgia and Louisiana, U.S.A.	400, **20**	100	laboratory colonies as sources; expression of *b* allele confirmed with microarray analyses

a *n*, number of individuals sampled; ***N***, number of polygyne colonies from which these individuals were sampled.

b number of sites from which individuals were sampled (number of nests not reported).

In order to directly examine the claim of LI08 that contradicts these previous findings, we conducted extensive sampling and testing of polygyne *S. invicta* colonies from College Station, Texas, a collection locality for one of the ostensibly polygyne samples in which workers reportedly failed to express the b allelic protein. Because the authors could not supply detailed collection locality information, we sampled 89 putative polygyne colonies from three sites dispersed around the city (see Table S1). Social organization of each colony initially was inferred in the field on the basis of nest distribution patterns, worker size distributions, and observation of multiple dealate queens (see Table 1 for terminology) [4], [39], [40]. However, because such information is not completely reliable, we definitively determined social organization for each colony as follows.

First, the mating status and reproductive development of up to four dealate queens per colony were determined; multiple mated queens with developed ovaries were found in 79 of the colonies. Next, for each of the remaining ten colonies from which only a single mated dealate queen was recovered, we determined genotype distributions for ten nestmate workers at six polymorphic allozyme loci. Worker genotype distributions in nine of these ten colonies were inconsistent with a simple-family (monogyne) colony structure in which all workers are the full-sister offspring of a single mother queen mated to a single (haploid) male [38], [41] (see Table 4); thus, we conclude that these were polygyne colonies from which we failed to recover more than a single reproductive queen during nest excavation. The final colony that we sampled (Col. 43) did contain workers whose genotype distributions implicated them as full sisters; moreover, the single mated dealate queen recovered possessed a multilocus genotype fully consistent with her being the mother of these workers (Table 4). Thus, the genotype composition of this colony is consistent with monogyne social organization. Finally, we assayed GP-9 of each sampled dealate queen from the 89 study colonies (*n* = 247). With the exception of the single queen from Col. 43, which exhibited only the single fast band characteristic of genotype *BB*, every other queen exhibited both bands, characteristic of genotype *Bb*. This is significant in that polygyne reproductive queens of S. *invicta* in the U.S.A. effectively always possess the *Bb* genotype, while monogyne queens bear the *BB* genotype [6]. In summary, we conclusively identified 88 of the College Station *S. invicta* colonies we sampled as polygyne, while one was determined to be monogyne.

**Table 4:** Genotype distributions at six polymorphic allozyme loci for inhabitants of ten College Station *S. invicta* colonies from which only a single mated dealate queen was collected.

	*Aat-2*			*Acoh-1*			*Acoh-5*			*G3pdh-1*			*Pgm-1*			*Pgm-3*		
	*100/100*	*100/144*	*144/144*	*82/82*	*82/100*	*100/100*	*93/93*	*93/100*	*100/100*	*40/40*	*40/100*	*100/100*	*96/96*	*96/100*	*100/100*	*89/89*	*89/100*	*100/100*
**Col. 23**																		
adult workers	7	3	0	0	4	5	0	3	5	***4***	***3***	***3***	***1***	***4***	***5***	***0***	***2***	***8***
**Col. 26**																		
adult workers	***12***	***1***	***0***	―	―	―	―	―	―	***7***	***7***	***4***	***0***	***1***	***13***	―	―	―
**Col. 40**																		
adult workers	***9***	***1***	***0***	0	0	10	0	0	10	***5***	***4***	***1***	0	0	10	0	10	0
**Col. 44**																		
adult workers	10	0	0	0	0	7	0	0	7	***2***	***4***	***3***	***0***	***2***	***8***	***1***	***6***	***3***
**Col. 51**																		
adult workers	***9***	***1***	***0***	0	3	7	0	4	6	***2***	***4***	***3***	***0***	***2***	***8***	***0***	***7***	***1***
**Col. 55**																		
adult workers	***9***	***1***	***0***	0	0	10	***0***	***1***	***9***	***3***	***4***	***1***	***0***	***2***	***8***	0	5	2
**Col. 61**																		
adult workers	***9***	***1***	***0***	0	2	7	***0***	***2***	***8***	***2***	***6***	***2***	***0***	***1***	***9***	***3***	***5***	***2***
**Col. 67**																		
adult workers	10	0	0	0	0	10	***1***	***5***	***4***	0	10	0	0	0	10	***2***	***6***	***2***
**Col. 83**																		
adult workers	***8***	***2***	***0***	***1***	***2***	***2***	0	0	5	***3***	***4***	***1***	0	3	5	0	10	0
**Col. 43**																		
dealate queen^a^			1			1			1		1			1				1
adult workers	0	10	0	0	0	10	0	10	0	7	3	0	0	7	3	0	0	10

Each locus possesses only two common alleles (three genotypes) in the U.S.A. [32]. Allele designations refer to relative electrophoretic mobilities of their products. Single-locus genotype distributions inconsistent with simple family (monogyne) colony social organization are shown in bold italics. Dashes indicate missing data.

a The single dealate queen recovered is presumed to be the mother queen of this monogyne colony.

**Figure 2:**
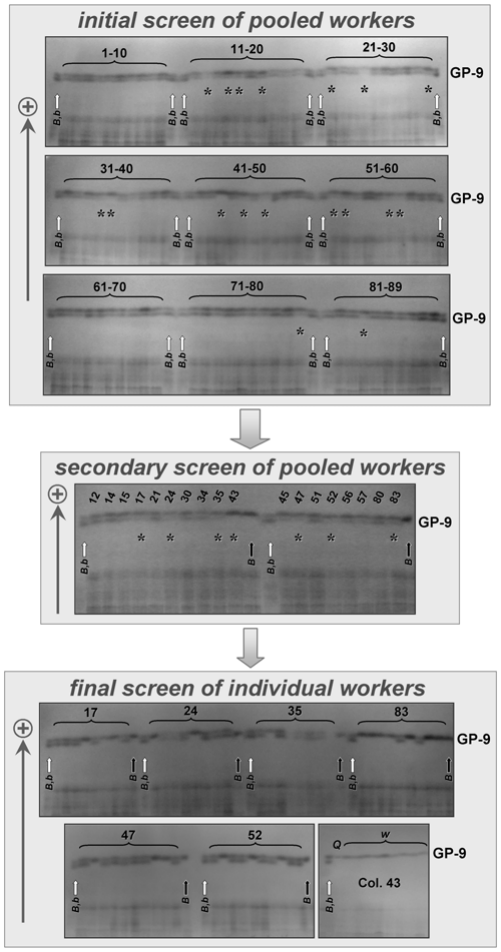
Results of sequential HSGE procedure for testing presence and expression of *Gp-9^b^* allele in adult *S. invicta* workers from College Station, Texas, U.S.A. In the initial screen, each lane contained the pooled protein extracts of 6–8 adult worker nestmates (head+thorax); the 89 colonies were arranged sequentially across the lanes in the three initial screen gels (identification codes for groups of colonies appear above lanes). Asterisks in the initial screen gels indicate colonies for which additional pooled extracts of another 6–8 nestmate workers were analyzed in the secondary screen (colony identification codes appear above lanes in the secondary screen gel). Asterisks in the secondary screen gel indicate colonies that were subsequently screened using individual extracts from 8–10 workers per colony (colony identification codes appear above each group of lanes in the final screen gels). In the small final screen gel, “Q” denotes the lane containing extract from the presumed mother queen from Colony 43, the only monogyne colony in the sample, whereas “w” denotes lanes containing worker extracts (fourteen additional Col. 43 workers not depicted were individually screened; all possessed genotype *BB*). For all gels, groups of lanes containing test material are bracketed by standards (white arrows, standards with both B and b allelic proteins; black arrows, standards with B allelic protein only).

We employed a sequential HSGE procedure to efficiently test for the presence and expression of the *Gp-9^b^* allele in workers from our College Station study colonies (Fig. 2). In an initial screen, protein extracts pooled from 6–8 mature adult workers per colony were assayed for the presence of the b allelic protein. Given that as few as 10% of workers in polygyne colonies may bear a *b* allele [11,12, K. G. Ross unpubl. data], there is a chance that such a worker would not be included in a pool of so few workers sampled from any single colony (or that so few *Bb* workers were included that the b protein band would be comparatively faint). Thus, we conducted a secondary screen of another pooled 6–8 workers on any colonies for which the b allelic protein band was not visible and pronounced on the initial gel. Finally, for the remaining few colonies for which the b band still was not pronounced on the secondary gel, we screened individual extracts from an additional 8–10 workers per colony. The results of this sequential procedure clearly demonstrate that each of our 88 verified polygyne colonies contained workers bearing and expressing the *Gp-9^b^* allele (Fig. 2). Moreover, the HSGE results described above demonstrate that every screened dealate queen from these colonies also bore and expressed the allele. Notably, there was no evidence for an expressed *b* allele among any of the workers from the single monogyne colony in our sample (Col. 43) screened singly (*n* = 24) or as pooled samples (*n* = 16) (Fig. 2), consistent with the invariant absence of such alleles previously reported in fire ant colonies of this social form. These results from our College Station *S. invicta* samples fully support previous findings that *Gp-9^b^* (or some other *b*-like allele) invariably is present in and expressed by some proportion of workers (as well as all queens) in polygyne colonies, thus contradicting a central claim of LI08.

The failure of LI08 to detect the b allele protein in three ostensibly polygyne samples (colonies), including one from the College Station area, can be attributed to any of several potential causes irrelevant to the proposed biological role of *Gp-9* in inducing polygyny. An obviously important, but often poorly appreciated, requirement for any study of the fire ant social forms is that the social organization of each study colony be correctly identified. Polygyne and monogyne colonies can be difficult to differentiate using the field criteria of worker size and nest dispersion patterns, especially when colonies of the two types are interspersed. The presence of multiple dealate queens is not in itself sufficient to reliably identify any particular colony as polygyne, because monogyne colonies can naturally contain numerous virgin, non-reproductive dealate queens under some circumstances [4], [42]–[44], and traumatic dealation can occur in association with nest excavation. To our knowledge, only four definitive criteria exist for distinguishing polygyne from monogyne colonies of *S. invicta* in the introduced U.S.A. range: i) presence of multiple dealate queens that are mated and possess developed ovaries [45], [46], ii) presence of multiple matrilines represented among the workers [47] (diagnostic except in the rare case of recent queen turnover in a monogyne colony [32]), iii) presence of diploid males [48], and iv) presence of triploid females [35]. Evaluation of the latter three criteria requires the application of genetic methods, with the result that these criteria rarely have been used outside of our circle of collaborators [e.g.], [ 9], [32], [38], [49]–[51]; evaluation of all four criteria requires that colony fragments be returned to the laboratory for study.

The four colonies studied in LI08 were not evaluated with respect to any of these criteria by the authors; instead, they point to the collectors of the colonies as having determined the social organization “at the time of the field collections” [27]. For the two Texas colonies, neither Dr. K. M. Heinz, who supplied the material to the authors, nor Dr. S. B. Vinson, who supplied the colonies to Heinz, were able to provide us with information on the provenance or chain of custody of the samples, nor could they tell us on what basis these samples were determined to have originated from polygyne colonies. Moreover, the paper's authors were unable to make any of their samples available to us for confirmation of social form. Thus, the social organization of the three colonies from Texas and from California for which LI08 failed to detect the b allele protein in workers must be considered uncertain (but see below for the California colony).

**Figure 3:**
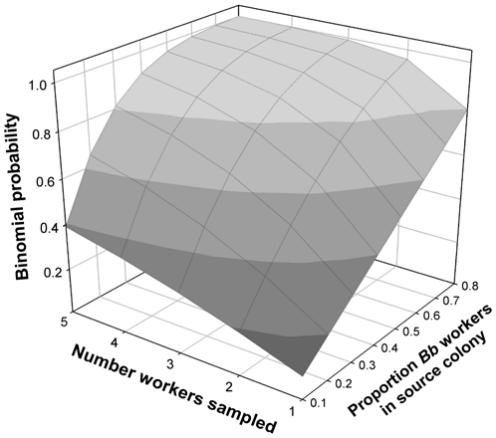
Binomial distribution probabilities of detecting *Gp-9^b^* transcript in sample of 28 clones distributed across samples of 1–5 worker ants obtained from colonies with differing *Gp-9* genotype proportions. The proportions of *Bb* workers in the source colony represent the range that exists in the wild in polygyne *S. invicta* in the U.S.A.

Even assuming that all the study colonies of LI08 were polygyne, their sampling procedures for cDNA sequence analyses may not have been adequate to detect the *b* allele product in any particular study colony. The authors sequenced no more than 28 *Gp-9* clones from mRNA extracts of workers from each of the four colonies. The probability of selecting at least one *Gp-9^b^* clone for sequencing out of 28 depends on several factors, including the number of sampled ants from which the clones were obtained and the genotype proportions in the source colony. Although it is not possible to confidently follow their sampling scheme based on the information provided, no more than five workers from each colony (and possibly as few as one for each of the Texas colonies) served as sources for the mRNA in LI08. Simple calculations of joint binomial probabilities show that if a source colony contains 30% or fewer *Bb* workers, then there is a good chance (20–60%) of missing a *Gp-9^b^* clone even with a sample of 28 clones distributed evenly across five workers (Fig. 3). If fewer workers are sampled, then the chances of selecting a *Gp-9^b^* clone decline precipitously. The frequency of workers bearing *Gp-9^b^* in polygyne colonies varies considerably in the wild, from around 80% to as low as 10% [11, K. G. Ross unpubl. data], and this lower proportion has been shown to be sufficient to elicit polygyne behavior in experimental laboratory colonies [11], [12]. (Virtually all *b*-bearing workers in polygyne colonies are *Bb* heterozygotes because of the apparent low viability of *bb* individuals [5], [31], [36].) We conclude from our probability calculations that the sampling effort of LI08 for their cDNA sequencing was not adequate to substantiate the existence of a previously unknown type of polygyne colony in which workers fail to express the GP-9^b^ protein.

**Figure 4:**
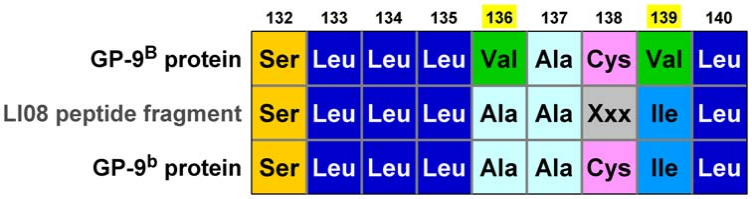
Amino acid sequences predicted for a peptide fragment of the B and b allelic variants of GP-9 from S. *invicta* in the U.S.A. and reported for the same fragment recovered from the California colony studied by LI08. The LI08 fragment was obtained by N-terminal sequencing of Lys-C digested GP-9. Amino acid positions for the native protein are indicated, with positions of fragment residues diagnostic for the B and b proteins in the U.S.A. highlighted. All b′ proteins from *S. invicta* (known only from the native South American range) feature a combination of Val^136^ and Ile^139^ residues [8]–[10].

Surprisingly, the claim of LI08 that GP-9^b^ protein was not expressed in workers in their California study colony, based both on cDNA sequencing and PAGE, is directly contradicted by results of a subsequent experiment they conducted to identify a potential phosphorylation site in GP-9. A reported peptide fragment recovered from adult workers of the California colony in the latter experiment contains two amino acid residues that are fully diagnostic for the GP-9^b^ protein of *S. invicta* in the U.S.A. (Fig. 4). Clearly, *Gp-9^b^* was expressed in workers of this colony.

From a technical standpoint, the California inconsistency raises doubts about the ability of the methods employed in LI08 to differentiate between the B and b variants encoded by *Gp-9*. Although the sampling issues discussed above must also factor into considerations of this inconsistency, there are causes for concern regarding the technical adequacy of the cDNA sequencing methods related to primer design. Specifically, the “gene specific” reverse primer RIFA-GP9br, presumably designed by LI08 with the aim of enriching amplification of the *b* transcript for subsequent sequencing, has the critical bases meant to confer allele binding specificity located near its 5′ end rather than within a few nucleotides of the 3′ end, so it is unlikely to have achieved the intended selective amplification [e.g., 52]. Compounding the difficulties in evaluating the sources of the California discrepancy, the authors did not indicate what nucleotide variation they used to distinguish between *B* and *b* alleles, and they did not deposit recovered nucleotide sequences in GenBank or another publicly available sequence depository. These issues are relevant in the following respect.

The charge-variant *b*-like alleles of *Gp-9*, including the *b* allele found in *S. invicta* in the U.S.A., represent only a portion of the diversity of *b*-like alleles associated with polygyny in *S. invicta* (Table 1) [8]–[10]. Given the few samples of introduced *S. invicta* for which *Gp-9* has been surveyed at the DNA sequence level (16 females from four localities [8]), it is conceivable that *b′* alleles, the class of *b*-like alleles lacking the charge-changing amino acid replacement of *Gp-9^b^*, occur in some areas of the U.S.A. in association with polygyny. Because such *b′* alleles encode products that are expected to be inseparable from the *B* allele product using charge-based electrophoretic methods, their presence in the California and Texas study colonies of LI08 presumably would be undetectable using native PAGE. Thus, the failure of LI08 to appreciate the full scope of variation at *Gp-9* and design assays accordingly could explain their inability to detect relevant GP-9 variation using PAGE in three of four ostensibly polygyne colonies. Lending credence to this scenario, the inferred native source population for *S. invicta* in the U.S.A., determined to reside in northeastern Argentina [53], contains both types of *b*-like alleles [9], [37]. Mitigating against it, a comprehensive survey of GP-9 in polygyne colonies from six widely dispersed sites in the U.S.A. failed to uncover evidence for electrophoretically cryptic *b′* alleles [32].

In order to learn if *b′* alleles occur along with *b* alleles in *S. invicta* in the U.S.A., we undertook an expanded survey of *Gp-9* variation, using as source material genomic DNA from 41 males obtained from both polygyne and monogyne colonies located in southeastern Mississippi, U.S.A. This area was chosen for sampling because of its proximity (<50 km) to Mobile, Alabama, the site of introduction of *S. invicta* to the U.S.A. [4], as well as the apparent genetic resemblance of populations in the area to the original founder population [32], [54]. Each of the 48 full-length *Gp-9* sequences we recovered is identical to one of the three *B* haplotypes (*B1*, *B2*, *B3*) or the single *b* haplotype (*b1*) described previously from the U.S.A. by Krieger and Ross [8] (see Table 1). In view of the minimal nuclear genomic differentiation that exists across the U.S.A. range of *S. invicta* [32], we believe that effectively all *Gp-9* variants in the introduced range have now been documented. Because none of these is a *b′* allele, it is unlikely that the presence of these electrophoretically cryptic *b*-like alleles, which typically occur in colonies to the exclusion of *b* alleles [31], contributed to the failure of LI08 to detect the b protein product using PAGE in three of their four study colonies.

In summary, a central claim of LI08, that a second type of polygyne colony of *S. invicta* exists in which adult workers (and reproductive queens) do not express the GP-9^b^ protein, is not supported by our detailed review of previous studies, our re-examination of the methods and results of LI08, or our expanded sampling and HSGE analyses of a source population for one of the LI08 study colonies. We conclude that the claim is almost certainly based on artifactual data resulting from improper determination of the social form of the study colonies, inadequacy of sampling, and/or technical deficiencies in the methods used to assay for GP-9^b^ transcripts and protein.

### GP-9 as a General Hemolymph Protein

The second central empirical claim of LI08, derived from their experiments with native PAGE, is that GP-9 protein is present in the hemolymph, and thus distributed throughout the body, of adult fire ant workers and queens. Gotzek and Ross [6] previously stated that “GP-9 protein routinely is extracted from whole-thorax or head homogenates of adult females, and it is abundant in thoracic hemolymph…, which suggests that it may circulate throughout the hemocoel”, a statement based largely on unpublished HSGE results such as shown in Figure 1. An important inference of this finding in the view of LI08 is that any primary role for GP-9 as a pheromonal chaperone or stimulator of neuronal activity within chemosensilla in the manner of the “gold standard” OBPs (Table 1) can be ruled out, because these confirmed chemosensory transducers are limited in their expression to chemosensilla (indeed, this is one of their defining features) [21], [55]. Nonetheless, LI08 demonstrate that GP-9 is present in the antennal club of fire ant workers [their Figure 8, see also 56], and other authors have suggested that OBPs with ubiquitous expression may have primary olfactory functions when they are expressed in olfactory organs [57; see also 58]–[61]. A somewhat stronger case that GP-9 does not function in a manner analogous to the “gold standard” OBPs can perhaps be made in light of the evidence presented by LI08 that it undergoes phosphorylation, an unknown form of post-translational modification among these proteins [60], [62, but see 63] [64]. Because comprehensive biochemical and expression data have been obtained for relatively few OBPs demonstrated to function specifically in chemosensory signal transduction [21], [55], [60], generalizations regarding their forms of post-translational modification and sites of expression should be viewed cautiously.

### GP-9 and the Genetic Regulation of Fire Ant Social Organization

The hemolymph distribution of GP-9 as well as the purported lack of expression of a b-like allelic protein in some polygyne *S. invicta* colonies led LI08 to conclude that “…it is highly unlikely that GP-9s are involved in olfactory mediation of social organization of the red imported fire ant.” In order to evaluate this very general conclusion, it is helpful to distinguish two separate, but interrelated, levels of explanation for the genetic basis of regulation of fire ant social organization, the specific biochemical and physiological pathways in which GP-9 functions and the manner in which variation in these functions affects production and perception of the stimuli modulating worker behaviors toward queens.

Regarding the biochemical level, the idea that the products of *Gp-9* may play primary chemosensory (olfactory or gustatory) roles similar to those proposed for the “gold standard” OBPs emerged from the convergence of two lines of evidence, the identification of GP-9 as a member of the OBP family [8] and the strong association of individual *Gp-9* genotypes of queens and workers with aggressive behaviors and responses known to be mediated by semiochemicals and central to worker control of colony queen number [6], [7], [16]. Initial enthusiasm for the idea that *Gp-9* plays a direct, exclusive role as a pheromone signal transducer has since given way to a more pluralistic outlook [6]. The more recent scenarios incorporate an increased recognition that chemosensory sensitivity and specificity is determined at multiple anatomical and physiological levels and sites [60], [65]-[67], with GP-9 potentially involved at any number of these. Among the possibilities considered by Gotzek and Ross [6] was that GP-9 may function “as a hemolymph carrier protein serving some primary function other than chemical communication, perhaps as a transporter of small hydrophobic endocrine factors”, a proposal echoed in the statement by LI08 that “…GP-9s are more likely involved in the transport of lipids or small ligands in the hemolymph…”. Indeed, the possibility that *Gp-9* plays no role whatsoever in the regulation of fire ant social organization, serving only as a functionally irrelevant marker for other genes with such roles, was considered explicitly in some early papers [5], [26], although it has since been discounted on the following grounds: i) the sex specificity and time course of GP-9 expression in queens and workers parallel patterns of semiochemical release and behavioral responses involved in regulating queen number, ii) positive selection has driven the molecular evolution of *Gp-9* (not linked genes) specifically in the context of the joint evolutionary origins of *b*-like alleles and polygyne social organization, and iii) the obligate association of *b*-like alleles with polygyny in *S. invicta* and related species has persisted in the face of recombination over evolutionary time scales [see 6], [9]. In light of these data, we feel that LI08's seeming conclusion that *Gp-9* is unlikely to be involved in any way in olfactory mediation of fire ant social organization is premature.

With regard to the behavioral level of explanation of *Gp-9* effects, a large obstacle to forming testable hypotheses is the lack of a detailed understanding of pheromonal communication systems of fire ants, this despite the fact that *S. invicta* is the best studied ant in this regard. Nonetheless, available general information on chemical communication in ants, as well as specific information on the relationship between *Gp-9* and fire ant social organization, certainly must be accommodated into theoretical frameworks if they are to be relevant to guiding empirical work on the *Gp-9* system (see Table 5). In overlooking or misappropriating much significant information, LI08 paint an unnecessarily confused, and in some respects irrelevant, picture of the pheromonal basis of regulation of queen number in fire ants. Queen primer pheromones of *S. invicta*, a focus of much attention in LI08, have been shown to attract workers to tend reproductive queens, prevent reproduction by alate queens in the nest, inhibit sexualization of female larvae, and, in monogyne colonies, mediate worker imprinting on the mother queen and execution of supernumerary reproductives [reviewed in 4]. Whether any of these diverse functions are mediated by the same releaser pheromone involved in the *Gp-9* phenomenology, as LI08 seem to believe, is unknown but seems doubtful. For instance, the need to disable queen imprinting effects when studying *Gp-9* effects [6], [7], as well as the lack of effect of queen reproductive status or ovarian development on queen acceptability once *Gp-9* genotype is taken into account [7], [68] — in contrast to a strong effect of these factors on many primer pheromone responses [4], [69]–[72] — suggest strongly that at least some of the above functions are served by pheromonal systems unrelated to *Gp-9*.

**Table 5:** Information on pheromonal communication in ants and on *Gp-9* and the behavioral regulation of fire ant colony social organization.

**Pheromonal communication in ants**
• Communication in ants typically is mediated by semiochemicals (pheromones) [2], [4].
▪ Releaser pheromones stimulate immediate responses of the nervous system that trigger immediate, specific behavioral responses.
▪ Primer pheromones alter the physiology through the endocrine or reproductive system, causing delayed behavioral responses.
• Ant pheromones serve many communication functions, including attraction, inhibition of reproduction, and recognition of nestmates, castes, or reproductive states [2].
• >40 exocrine glands are known to produce pheromones in ants [2], [67].
• An enormous diversity of compounds is used in ant pheromones; optimal responses often are achieved through specific blends of compounds rather than unique compounds [2], [67].
• Each pheromonal component can have both independent and synergistic effects, as exemplified by *S. invicta* trail pheromone [e.g., 80], and particular pheromones (or constituent components) often are used in multiple roles in different contexts.
• Pheromonal signals can be fine-tuned by auxiliary tactile or auditory cues [e.g., 81].
• *Solenopsis invicta* appears to possess 20 or more pheromone systems, although the anatomical, chemical, and behavioral independence of most remains unclear [4], [71].
***Gp-9*** ** and behavioral regulation of fire ant social organization**
• The number and identity of reproductive queens in a colony is under the collective control of workers, which tolerate and nurture queens judged to be acceptable as supernumerary or replacement reproductives and destroy the remainder.
• Regulation of fire ant colony queen number involves an interaction of worker and queen *Gp-9* genotypes that implicates the gene product as affecting both pheromone production and perception.
▪ Colonies containing only homozygous *BB* workers (monogyne colonies) accept single *BB* replacement reproductive queens but do not tolerate queens bearing the *b* allele.
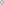 Such replacement queens are acceptable only if the colony has been queenless for several days.
▪ Colonies containing workers with the *b* allele (polygyne colonies) accept multiple reproductive queens also bearing this allele but do not tolerate *BB* queens.
• Aggression toward queens lacking allele *b* in polygyne colonies is perpetrated mainly by workers that possess this allele.
• Worker aggression toward pre-reproductive queens lacking allele *b* escalates in polygyne colonies when these queens are between a few days and two weeks of adult age, coincident with the onset and intensification of their GP-9 expression.
• Worker aggression toward queens lacking allele *b* in polygyne colonies is released by a queen signal that resides on the cuticle.
• Worker discrimination among queens on the basis of *Gp-9* genotype is not influenced by the social environment previously experienced by workers or queens or by the state of queen reproductive development (penetrance of *Gp-9* is high).
• Worker discrimination among queens on the basis of *Gp-9* genotype can be dampened if workers are imprinted on a single queen or extinguished altogether if workers are held queenless for prolonged periods (several days or more).

Information in the second list pertains specifically to *S. invicta* in the U.S.A. and is based on summaries in [6].

LI08 prominently cite as background to their work two studies that were conducted on such presumably unrelated systems and that did not attempt to account for *Gp-9* effects [69], [73], thus making their relevance to the higher-level role of *Gp-9* in regulating colony queen number questionable. LI08 then compound this problem by mischaracterizing the state of knowledge of the *Gp-9* system. Specifically, they mistakenly attribute the social form of source and recipient colonies, rather than the *Gp-9* genotypes of introduced queens and of recipient colony workers, as the major determinants of the outcomes of decisive behavioral assays (see Table 5). This oversight stems at least in part from their apparent reliance on an outdated secondary account of fire ant biology [74], information in which has been superseded by recent authoritative reviews of fire ant social biology [4], [6] as well as comprehensive behavioral studies aimed specifically at testing the relative roles of *Gp-9* genotype and other factors in the regulation of colony queen number [7], [11], [12].

## Discussion

The *Gp-9* system in fire ants has emerged as an important model for studying the genetic basis of social evolution in insects as well as a rich source of information relevant to other evolutionary phenomena. While a great deal has been learned about this system over the past decade, enormous gaps remain in our knowledge of the functional role of GP-9 protein, the biochemical and physiological pathways in which the protein functions, the identity of other genetic and biochemical components of these pathways with which *Gp-9* interacts, the pheromones involved in mediating worker regulation of queen number, and the specific behaviors by which this regulation is achieved. A significant challenge to researchers wishing to help fill this knowledge gap is that the effects of *Gp-9* are played out within a complex social system, and familiarity with this system as well as other basic elements of fire ant biology is key to designing, implementing, and interpreting meaningful studies. Fire ants have been the focus of numerous studies since the introduction of *S. invicta* to the U.S.A. in the 1930s, with the result that relevant information is scattered across a vast literature of uneven quality. Several reviews that cover aspects of fire ant biology pertaining to the *Gp-9* system (generally above the molecular/biochemical level) provide useful starting points in sorting through and evaluating such information [4], [6], [40].

The recent contribution of Leal and Ishida [27; LI08] concludes that the existence of polygyne (multiple queen) *S. invicta* colonies that lack inhabitants expressing GP-9^b^ protein, as well as the presence of GP-9 protein in the hemolymph, indicate that the *Gp-9* gene is unlikely to be involved in olfactory mediation of fire ant social organization. In reviewing existing literature, analyzing the methods and results of LI08, and collecting new data from one of their study sites, we conclude that their claim that polygyny can occur in the absence of expression of *b*-like alleles (*Gp-9^b^* in the case of *S. invicta* in the U.S.A.) is unfounded. Moreover, we conclude that available information on insect OBPs (the protein family to which GP-9 belongs), on the evolutionary/population genetics of *Gp-9*, and on pheromonal/behavioral regulation of fire ant colony queen number cannot be used to support their conclusion that GP-9 plays no role in the chemosensory-mediated communication that underpins worker regulation of social organization. Many of the problems inherent in the LI08 study might have been avoided had the authors drawn appropriately on the pertinent literature on pheromonal communication in social insects, fire ant social organization, and the *Gp-9* system.

In many respects the debate over the specific biochemical/physiological function of GP-9 raised by LI08 has become sterile and unproductive. While existing data indicate that many insect OBPs likely have far more diverse roles than acting simply as primary chemosensory transducers in the manner of the “gold standards,” only rigorous studies of the molecular interactions, specific ligands, and sensillar compartmentalization of GP-9 protein will definitively demonstrate its specific functions (but see [65] for a corrective on even this view). Determination of the higher-level role of the protein in modulating individual and colony-level behaviors will be even more challenging, given our profound ignorance of fire ant pheromonal systems and the behaviors involved in regulation of colony queen number. In this vein, we re-iterate the caveat of Gotzek and Ross [6] that progress in dissecting the higher levels of explanation of the *Gp-9* phenomenon is complicated by the fact that colony organization is a social phenotype, the collective result of reciprocal communication and behavioral interactions among hundreds or thousands of individuals that differ in their genetic composition, morphology, age, and experience. Accounting for this complexity while retaining a natural context in the design of relevant pheromonal and behavioral assays is a necessary but difficult task for future studies.

## Materials and Methods

### HSGE Analyses of *S. invicta* from College Station, Texas

We collected samples from 89 suspected polygyne *S. invicta* colonies at three sites in College Station, Texas (see Table S1), a locality from which one of the four study colonies of LI08 originated (their Texas A&M colony). The sampled colonies were the only ones suspected to be polygyne, based on initial field criteria [39], [40], out of several hundred colonies inspected at the three sites. The focal nests were excavated and samples of 20–100 workers were aspirated from each; dealate queens were collected opportunistically. All samples were immediately placed on liquid nitrogen in the field and then held in a −80°C freezer upon return to the laboratory. Social organization initially inferred for each colony in the field was definitively determined in the following way. The mating status and reproductive development of up to four dealate queens per colony were determined by dissecting the spermathecae and ovaries of 247 queens from the 89 colonies; queens were judged to be mated when the spermatheca was white and opaque, and they were judged to be reproductively active when the ovaries contained multiple fully developed eggs [e.g., 46]. For the subset of ten colonies in which only a single mated queen was recovered, we determined genotype distributions for ten randomly chosen nestmate workers at six polymorphic allozyme loci using HSGE coupled with specific histochemical staining [75]. Monogyne fire ant colonies display a simple family structure in which all workers are the full-sister offspring of a single mother queen mated to a single (haploid) male [38], [41]; therefore, genotype distributions inconsistent with such structure provide strong evidence of polygyny. For bi-allelic loci, such distributions feature three unique genotypes, two unique homozygous genotypes, or two unique genotypes that depart from a 1∶1 ratio (determined using binomial tests).

Expression of *Gp-9* and variation in the protein products were studied in the College Station samples using the HSGE methods detailed in DeHeer et al. [28]. Soluble proteins were extracted from the head+thorax of individual workers or pools of workers by macerating the material in 50 mM tris-HCl buffer solution (15 µL/individual). GP-9 bands were stained and visualized as described in DeHeer et al. [28]. GP-9 standards derived from individuals of known *Gp-9* genotype (*BB* and *Bb*) were included in multiple lanes of every gel.

With the following exceptions, these same procedures were used to generate the gel images shown in Figure 1. To detect GP-9 in the hemolymph, the fluid was directly expressed onto paper wicks soaked in tris-HCl buffer by applying gentle pressure to the anterior opening of decapitated thoraces; the wicks were then loaded into gels and subjected to electrophoresis. Protein extracts for pooled samples of eggs, larvae, and adult males were concentrated in a vacuum concentrator prior to loading into gels for electrophoresis.

### Sequence Analyses of *Gp-9* in *S. invicta* from Southeastern Mississippi

Nucleotide sequences of *Gp-9* were obtained from the genomic DNA of 41 *S. invicta* males collected around the cities of Hurley and Pascagoula, Mississippi; sources of these samples were 15 polygyne and 26 monogyne colonies [see 32 for details]. Sequence data were generated as described in Krieger and Ross [8] and Gotzek et al. [9]. Two sequences were recovered from each of seven heterozygous diploid males, while only a single sequence was recovered from the remaining 34 haploid or homozygous diploid males. The resulting 48 sequences were visually checked and aligned using the program Lasergene v8.0 (DNAStar), and are deposited in GenBank under accession numbers GU086668–GU086715.

### Binomial Probability Calculations

We evaluated the sampling sufficiency for the cDNA sequencing that LI08 employed to determine if workers present in a colony express *Gp-9^b^*. We used binomial distributions to calculate the joint probabilities of detecting a b transcript given that 28 clones were obtained from 1–5 sampled workers from single source colonies containing different proportions of *BB* and *Bb* workers. We used genotype proportions in the source colonies that span the range observed in polygyne *S. invicta* colonies in the U.S.A. [11, K. G. Ross unpubl. data]. These colonies were assumed to lack workers with the *bb* genotype — because of their apparent low viability, such workers are rarely found [5], [36]. We further assumed that the 28 clones were distributed evenly across the sampled workers.

## Supporting Information

Table S1 Collection information for 89 *S. invicta* colonies sampled from College Station, TX in March, 2009.
(0.02 MB XLS)
